# Socioeconomic disadvantages and minority race correlate with worse outcomes following shoulder arthroplasty: a systematic review

**DOI:** 10.1016/j.xrrt.2025.04.011

**Published:** 2025-05-21

**Authors:** Cailan L. Feingold, Eric H. Lin, Justin W. Zheng, Ashley Mulakaluri, Joseph N. Liu

**Affiliations:** Department of Orthopaedics, Keck School of Medicine of USC, Los Angeles, CA, USA

**Keywords:** Sociodemographics, Race, Shoulder arthroplasty, Outcomes, Socioeconomic status, Social determinants of health

## Abstract

**Background:**

It has been established that patient sociodemographics including race, socioeconomic status, insurance status, and education can impact outcomes following orthopedic procedures. This study aims to investigate how these patient sociodemographics impact outcomes following shoulder arthroplasty.

**Methods:**

Pubmed, Scopus, and Embase were queried for terms related to shoulder arthroplasty and patient demographics for studies published between 2000 and Jan 30, 2025. Studies were included if they were clinical studies of total shoulder arthroplasty (including reverse, anatomic, and hemiarthroplasties) investigating how 1 of four patient sociodemographics (insurance status, race, socioeconomic status, and education) influence either clinical or patient-reported outcomes. Study quality was evaluated using the Methodological Index for Non-Randomized Studies (MINORS) criteria. Study results were recorded descriptively.

**Results:**

Twenty-two studies met the inclusion criteria. Twelve (54.5%) studies investigated clinical outcomes alone, 6 (27.3%) studies investigated PROMs alone, and 4 (22.2%) studies investigated both. The sociodemographics studied were insurance status in 9 (40.9%) studies, race in 7 (31.8%) studies, socioeconomic status in 14 (63.6%) studies, education in 2 (9.1%) studies, and employment and marital status both in 1 (4.5%) study. Eight (88.9%) studies found public insurance to negatively affect at least 1 outcome, 5 (71.4%) found minority races to be associated with worse outcomes, 9 (64.2%) found socioeconomically disadvantaged patients to be associated with poor outcomes, 2 (100%) studies found educational disparity to influence outcomes negatively, and 1 (100%) study found an association with employment. Overall, 17 (77.3%) studies found a significant association between at least 1 of the five sociodemographics of focus and outcomes following shoulder arthroplasty.

**Conclusion:**

The majority of studies investigating the influence of insurance status, race, socioeconomic status, education, and employment on shoulder arthroplasty outcomes found significant associations. Patients using public insurance, who are minorities and who are socioeconomically or educationally disadvantaged, should all be identified by physicians as being at higher risk of poor outcomes. Physicians should partner with these patients to counsel them accordingly and to identify opportunities to improve their chances of success.

Shoulder arthroplasty is an effective treatment option for patients with refractory shoulder pain that has failed conservative treatment. The two primary options are anatomic total shoulder arthroplasty (aTSA) and reverse total shoulder arthroplasty (rTSA). Both reverse and anatomic shoulder arthroplasty have proven beneficial for both young, active patients and older, more sedentary patients.^2^^,^^15^^,^^18^^,^^34^ This likely explains why reverse and aTSA incidence has increased substantially since 2012.^3^ Hemiarthroplasties, also known as partial shoulder replacements, replace only the humeral head with an implant.^12^

Outcomes following surgery are influenced by multiple factors, but 1 crucial element often overlooked is the unique sociodemographic profile of the individual patient. Patient race, insurance status, income, and education are all sociodemographic variables that affect outcomes in orthopedic surgery.^21^^,^^24^^,^^28^^,^^41^ More specifically, this has also been shown within the arthroplasty literature. In patients undergoing total hip and knee arthroplasties, low socioeconomic status is associated with worse outcomes,^33^ as is lower education level,^50^ minority race,^36^ and public insurance.^7^

Much of the existing research on the role of patient sociodemographics in outcomes following arthroplasty is isolated to the hip and knee arthroplasty procedures. Given the increasing incidence of total shoulder arthroplasty and the variety of patients undergoing the procedure, understanding the association between outcomes and patient sociodemographics is essential. This study aims to evaluate how patient race, insurance, socioeconomic status, and education influence outcomes following both reverse and aTSA using a systematic review.

## Methods

### Study selection

This study was conducted following the Preferred Reporting Items for Systematic Reviews and Meta-Analyses guidelines. Pubmed, Scopus, and Embase were queried using the search: (“shoulder arthroplasty” OR “shoulder replacement”) AND (“socioeconomic” OR “sociodemographic” OR race OR income OR education∗). The search period was from January 1, 2000, to January 30, 2025. Studies were included if they were (1) clinical studies of shoulder arthroplasty (including rTSA, aTSA, and hemiarthroplasties) and (2) investigated how patient sociodemographics influence either clinical or patient-reported outcomes. Excluded studies were case reports, systematic reviews, and meta-analyses. Article screening was performed by two independent authors (C.L.F and E.H.L.), first by title and abstract and then by full text. Study screening was performed in Covidence (Melbourne, Australia) which identified and removed any duplicates prior to manual screening.

A quality assessment of all included studies was carried out using the Methodological Index for Non-Randomized Studies (MINORS).^45^ For noncomparative studies the maximum MINORS score indicating high quality is 16 and for comparative studies the maximum score is 24.

### Data collection/analysis

General study characteristics, including year of publication, journal, and level of evidence (LOE), were collected. The sociodemographics being evaluated were recorded, as well as the patient-reported outcome measures (PROMs) or clinical outcomes that served as endpoints for the studies. Authors collected study results and compiled them in Excel (Microsoft, Redmond, WA). Results were reported descriptively.

## Results

**Figure 1:**
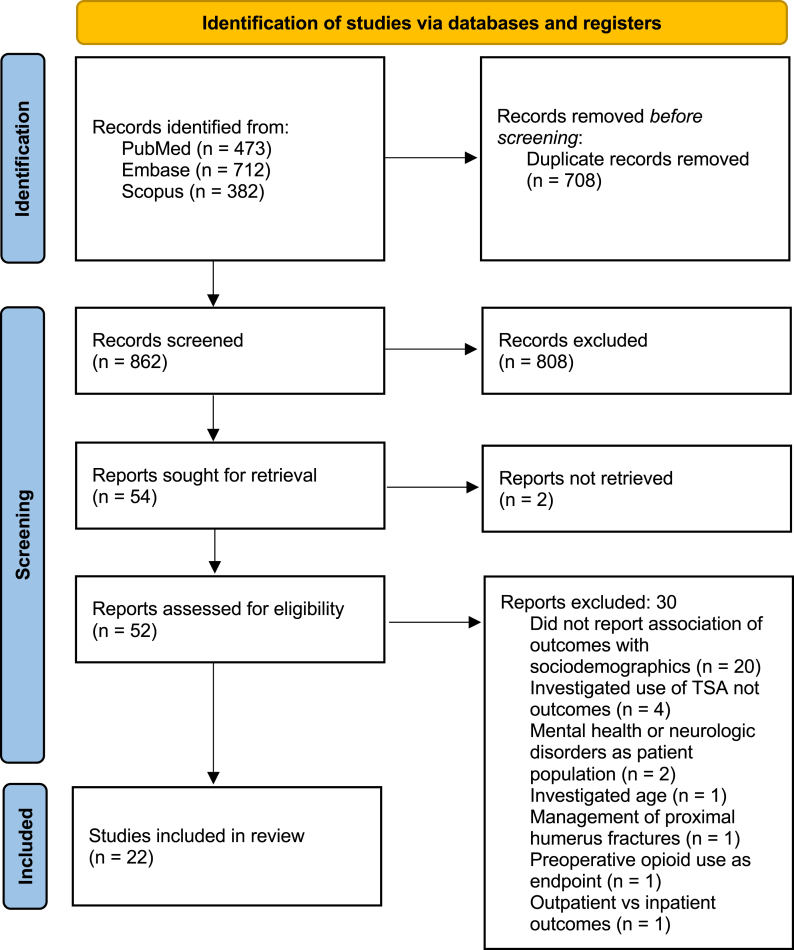
PRISMA diagram detailing search strategy and screening results. *PRISMA*, Preferred Reporting Items for Systematic Reviews and Meta-Analyses.

Initial search results yielded 712 studies and a total of 22 studies were included for analysis (Fig. 1).^5^^,^^10^^,^^11^^,^^13^^,^^14^^,^^22^^,^^25^^,^^26^^,^^30^^,^^35^^,^^37^^,^^38^^,^^42^, ^43^, ^44^^,^^46^^,^^48^^,^^49^ The average MINORS score for the 5 (22.7%) noncomparative studies was 9.8 ± 2.2 and the average MINORS score for the 17 (77.3%) comparative studies was 15.8 ± 1.4 (Table I). In terms of LOE, no studies were of LOE I or II, 12 (54.5%) were LOE III, and 10 (45.5%) were LOE IV. The included studies were published between 2015 and 2025, with 19 (86.4%) published during or after 2020. Six (27.3%) studies investigated the association of sociodemographics with PROMs alone, 12 (54.5%) investigated the association of sociodemographics with clinical outcomes alone, and 4 (18.2%) investigated these associations with both PROMs and clinical outcomes. All but two (90.1%) investigated both aTSA and rTSA. One investigated rTSA only,^42^ and 1 investigated aTSA only.^49^ Three studies included hemiarthroplasties.^5^^,^^19^^,^^25^

**Table I:** MINORS quality assessment.^45^

Authors	1	2	3	4	5	6	7	8	9	10	11	12	Noncomparative sum	Comparative sum
Smucny et al^46^	2	2	1	2	0	1	1	0	-	-	-	-	9	
Waldrop et al^48^	2	2	1	2	0	2	0	0	2	2	1	2		16
Singh et al^43^	2	2	1	2	0	1	0	0	-	-	-	-	8	
Garcia et al^14^	2	2	1	2	0	1	1	0	2	2	1	2		16
Landsown et al^25^	2	2	2	2	0	2	1	0	2	2	1	2		18
Sabesan et al^37^	2	2	1	2	0	2	0	0	2	2	1	2		16
Moverman et al^30^	2	2	2	2	0	2	0	0	1	2	1	2		16
Cutler et al^10^	2	2	1	2	0	1	1	0	2	2	1	2		16
Raso et al^35^	2	2	1	2	0	2	1	0	2	2	2	2		18
Farronato et al^11^	2	2	1	2	0	1	0	0	2	2	1	2		15
Silva et al^42^	2	2	2	2	0	2	1	0	-	-	-	-	11	
Mandalia et al^26^	2	1	1	2	0	1	1	0	2	2	1	2		15
Khlopas et al^22^	2	2	1	2	2	2	2	0	-	-	-	-	13	
Gammel et al^13^	2	1	1	2	0	1	1	0	2	2	2	2		16
Wu et al^49^	2	2	1	2	0	2	0	0	2	2	2	2		17
Castle et al^5^	2	2	1	2	0	1	2	0	1	1	2	2		16
Schell et al^38^	2	1	1	2	0	1	1	0	1	1	1	2		13
Singh et al^44^	2	1	1	2	0	1	1	0	-	-	-	-	8	
Jensen et al^19^	2	1	1	2	0	2	1	0	2	2	0	2		15
Morgan et al^29^	2	2	1	2	0	2	0	2	2	2	2	2		17
Thomas et al^47^	2	2	1	2	0	0	0	0	2	2	0	2		13
Bethell et al^4^	2	2	1	2	0	2	0	0	2	2	0	2		15

*MINORS*, Methodological Index for Non-Randomized Studies.

1. Clearly stated aim. 2. Inclusion of consecutive patients. 3. Prospective collection of data. 4. Endpoints appropriate to aim of study. 5. Unbiased assessment of study endpoint. 6. Follow-up period appropriate to aim of study. 7. Loss to follow-up less than 5%. 8. Prospective calculation of study size. 9. Adequate control group. 10. Contemporary group. 11. Baseline equivalence of groups. 12. Adequate statistical analysis. Score of 0 indicates not addressed, score of 1 indicates addressed but insufficient, score of 2 indicates addressed and sufficient.

### Overall

Seventeen (77.3%) studies found that the insurance payor, race, socioeconomic status, education, and employment of patients undergoing shoulder arthroplasty affected outcomes (Table II). Only four (22.2%) studies found no outcome of focus to be affected by patient sociodemographics.

**Table II:** Sociodemographic variable(s) and outcome(s) of interest and significant findings of each study.

Study	I.S.	R.E.	S.E.S.	E.S.	Emp.	M.S.	PROM(s)	Clinical outcome(s)	Major findings
Gammel et al^13^	X							X	1. Medicaid patients had higher odds of all-cause complications, readmission, and mortality within 180 d 2. Medicaid patients had higher odds of implant-related complications 3. Medicaid patients had increased mean cost and increased mean length of stay
Khlopas et al^22^			X				X		1. Patients with higher area deprivation index (ADI) were less likely to achieved minimal clinically important differences (MCID) and patient acceptable symptomatic state (PASS) in most PROMs 2. Higher ADI associated with worse PROMs preoperatively and postoperatively, as well as worse improvement in PROMs postoperatively 3. ADI did not affect implant survival
Mandalia et al^26^			X					X	1. Median social deprivation index (SDI) was higher in patients who developed complications within 12 mo postop 2. Patients with higher SDI had higher rates of readmission, dislocation, humeral fractures, deep vein thrombosis, wound complications at 1-, 3-, and 12- mo postop 3. Patients with higher SDI had higher rates of pulmonary embolism at 3-mo postop
Silva et al^42^			X				X	X	1. Patients from low socioeconomic class experienced significant improvements in PROMs and ROM 2. All returned to daily life activities
Castle et al^5^	X	X	X				X		1. Black patients had significantly worse PROMIS-UE and PROMISE-PI scores than caucasian patients 2. Patients from the lowest median household income had significantly worse PROMIS-UE and PROMISE-PI scores 3. Patients with government/public insurance had significantly worse PROMIS-UE and PROMISE-D scores
Schell et al^38^	X	X	X					X	1. African American and Hispanic patients had increase length of stay 2. Hispanic patients had increased risk of complications 3. Medicare patients had higher risk of readmission, revision, discharge to facility, and hospital stay extension 4. Patients from the lowest income quartile had increased risk of hospital stay extension, complications, and readmissions 5. Patients from the second and third lowest income quartiles had increased risks of complications
Farronato et al^11^			X					X	1. Patients from distressed communities are more likely to experience unplanned readmissions than those from more prosperous communities 2. Patients from distressed, at-risk, midtier, and comfortable communities exhibit increased health-care utilization postoperatively
Raso et al^35^			X	X				X	1. Economic disparity was associated with increased major complications, minor complications, ED visits at 90 d postoperatively and risk of aseptic loosening, instability, and revision at 1 yr postoperatively 2. Educational disparity associated with minor complications, ED visits, and instability 3. Environmental and social disparities not significantly associated with outcomes
Cutler et al^10^		X	X					X	1. Race and income quartile are not predictive of need for revision
Moverman et al^30^			X				X		1. No difference in preoperative or postoperative PROMs based on socioeconomic disadvantage
Sabesan et al^37^	X						X	X	1. No difference in postop PROMs between medicaid and non-Medicaid patients 2. No difference in ROM between medicaid and non-Medicaid patients 3. All scores improved postoperatively for both groups
Lansdown et al^25^	X						X	X	1. Medicaid patients had worse preoperative and postoperative PROMs 2. Medicaid patients had worse follow-up
Wu et al^49^	X						X	X	1. Worse PROMs in workers compensation patients than in nonworkers compensation patients 2. Workers compensation patients did not demonstrate a difference in revision rates
Garcia et al^14^		X						X	1. Hispanics had lower risk of revision compared to Caucasian patients 2. No difference in revision risk for Asian or Black patients compared with Caucasian patients 3. No racial differences in readmission risk 4. Black patients had higher risk of ED visits postop
Singh et al^43^	X		X					X	1. Government insurance (Medicare and Medicaid) patients were more likely to have a length of stay greater than 2 d, to be discharged to rehabilitation facility, and to undergo transfusion 2. Medicaid patients had higher risk of fracture 3. Medicare patients had higher risk of infection 4. Patients from the lowest income quartile had higher risk of length of stay greater than 2 d, undergoing transfusion, hospital charges higher than median, and revision
Waldrop et al^48^	X		X				X		Socioeconomically disadvantaged (patients on public insurance under the age of 65) demonstrated significantly worse PROMs before and after surgery
Singh et al^44^		X						X	1. A higher proportion of black patients than white patients had a hospital length of stay that was longer than the median 2. No difference in race for discharge to medical facility
Smucny et al^46^	X							X	1. More inpatient surgical site infections were seen in patients with Medicaid insurance
Jensen et al^19^				X	X	X	X		1. unemployed patients had significantly lower PROMs compared to low-level jobs, high-level jobs, and retired patients 2. Low education was associated with lower PROMs compared with medium and high education 3. Income and marital status had no associations with PROMs
Morgan et al^29^			X				X		1. PROMs for all groups improved significant 2. Low and moderate SES scored lower on PROMs than high SES
Thomas et al^47^		X	X					X	1. Hispanic patients were significantly less likely to undergo revisions than white patients. 2. Black patients were significantly more likely to undergo revision due to implant loosening, osteolysis, or broken implants than white patients but less likely to undergo revision due to dislocation or fracture 3. Revision risk was similar between socioeconomic groups
Bethell et al^4^		X	X					X	1. low SES had significantly higher rate of 90-d readmissions 2. High SES patients had higher health-care contact in 90 d follow-up period (median of 16 visits vs. 13 and 10 in middle and low) 3. Strong association between 90-d readmissions and neighborhood deprivation 4. Race not associated with 90-d readmissions 5. Higher SES patients had higher costs within 90 d

*IS*, insurance status; *RE*, race or ethnicity; *SES*, socioeconomic status; *ES*, education status; *Emp.*, employment status; *MS*, marital status; *PROM*, Patient-Reported Outcomes Measure; *ED*, emergency department; *PROMIS-UE*, patient-reported outcomes measurement information system upper extremity; *PROMIS-PI*, patient-reported outcomes measurement information system pain interference.

### Insurance

Nine (50%) studies evaluated how insurance payors affected outcomes in shoulder arthroplasty.^5^^,^^13^^,^^25^^,^^37^^,^^38^^,^^43^^,^^46^^,^^48^^,^^49^ Eight (88.9%) studies found differences in outcomes based on insurance status. In 2 (22.2%) studies, PROMs were found to be worse postoperatively for patients with both Medicare and Medicaid.^5^^,^^48^ Patients with Medicaid demonstrated worse PROMs preoperatively and postoperatively compared with both private and Medicare patients in another (11.1%) study.^25^ One (11.1%) study found no differences in postoperative PROMs between Medicaid and non-Medicaid patients.^37^

In terms of clinical outcomes, patients on public insurance options had worse clinical outcomes, including infections, fractures, readmission, increased length of stay, revision, and mortality, in 4 (80%) studies.^13^^,^^38^^,^^43^^,^^46^ However, 1 (11.1%) study found no difference in range of motion (ROM) between Medicaid and non-Medicaid patients.^37^

One (11.1%) study investigated how workers' compensation impacted outcomes for patients undergoing aTSA.^49^ For workers' compensation patients, PROMs were worse than their nonworkers’ compensation counterparts.^49^ In terms of clinical outcomes, though, Wu et al found no difference in revision rates between workers' compensation patients and nonworkers’ compensation patients.^49^

### Race/ethnicity

Race/ethnicity's impacts on shoulder arthroplasty outcomes were investigated in 7 (31.8%) studies.^4^^,^^5^^,^^10^^,^^14^^,^^38^^,^^44^^,^^47^ Five (71%) studies found at least 1 race-based difference in outcomes.^5^^,^^14^^,^^38^^,^^44^^,^^47^ Garcia et al found that Black patients demonstrate significantly more ED visits than White patients and that Hispanic patients are at a lower risk of revision than White patients.^14^ Thomas et al similarly found Hispanic patients to be at lower risk of revision than White patients.^47^ Schell et al demonstrated that Black and Hispanic patients are more likely to experience longer lengths of stay and Hispanic patients are at increased risk of complications.^38^ Thomas et al demonstrated that Black patients were more likely to undergo revisions due to implant loosening, osteolysis, and broken implant than White patients, but were less likely to undergo revision due to dislocation or fracture.^47^ Similarly, Singh et al found an association between Black race and longer length of stay.^44^ Four (57%) of the studies investigating race found outcomes with no race-based differences, including discharge to a medical facility,^44^ readmission,^14^ and revision.^10^^,^^47^

Only 1 (20%) study investigated racial impact on PROMs and found that they are worse among Black patients compared with White patients.^5^

### Socioeconomics

Fourteen (63.6%) studies evaluated how socioeconomic status and income affected outcomes.^4^^,^^5^^,^^10^^,^^11^^,^^19^^,^^22^^,^^26^^,^^29^^,^^30^^,^^35^^,^^38^^,^^42^^,^^43^^,^^47^ At least 1 outcome was found to be influenced by socioeconomic status in 9 (64.3%) studies.^4^^,^^5^^,^^11^^,^^22^^,^^26^^,^^29^^,^^35^^,^^38^^,^^43^ For clinical outcomes, lower socioeconomic status was associated with increased risks of revision, ED visits, 90-day readmissions, and major complications, including deep vein thrombosis, pulmonary embolism, and humeral fractures.^4^^,^^11^^,^^26^^,^^35^^,^^38^^,^^43^ However, Thomas et al found revision risk to be similar between socioeconomic groups.^47^ Cutler et al found that income quartile was not predictive of revision, and Khlopas et al similarly found that a higher area deprivation index (ADI) was not associated with implant survival.^10^^,^^22^ Silva et al did not compare outcomes between socioeconomic statuses but did find that in a series of low-income patients undergoing rTSA, all experienced significant improvement in ROM with a low risk of complications.^42^

In terms of PROMs, socioeconomically disadvantaged patients also demonstrated worse PROMs in 3 (23.1%) studies.^5^^,^^22^^,^^29^ However, Moverman et al found no difference in PROMs based on socioeconomic status.^30^ PROMs improved significantly for all low-income patients undergoing rTSA in Silva et al^42^ Jensen et al demonstrated no differences in PROMs based on patient income.^19^

### Education

Two (9.1%) studies considered how educational disparities influenced outcomes.^19^^,^^35^ Raso et al found that educational disparity increased the risk of minor complications, ED visits, and instability.^35^ Jensen et al demonstrated that patients with lower education levels exhibited lower PROM scores than those with medium-level and high-level of education.^19^

### Employment

One (4.5%) study investigated how employment status affects PROM scores following TSA.^4^ Unemployed patients exhibited significantly lower PROMs compared with those with low-level jobs, high-level jobs, and retired patients.^19^

## Discussion

The majority of studies found that patient sociodemographics, including insurance, race, socioeconomics, education, and employment were associated with clinical and patient-reported outcomes following shoulder arthroplasty. Insurance and socioeconomic status were the two factors investigated most frequently. Socioeconomic status was the most common variable to be associated with shoulder arthroplasty outcomes, with lower income or worse ADI being affiliated with worse outcomes. Insurance status was the next most frequent variable related to worse outcomes following shoulder arthroplasty. The unique sociodemographic profiles of individual patients may put them at risk of experiencing worse outcomes and should be used to identify higher-risk patients.

Insurance payors were associated with outcomes in nearly all of the studies that investigated this relationship. This may be due to how insurance status affects access to care. For example, in access to arthroplasty procedures alone, insurance influences time to operation and incidence. Medicaid patients undergo fewer total hip arthroplasties (THAs) and deal with longer wait times for both THA and total knee arthroplasty than commercially insured and Medicare patients.^6^^,^^23^ Longer wait times may factor into the significantly worse preoperative PROMs in shoulder arthroplasty patients identified by multiple studies here.^5^^,^^25^ Following shoulder arthroplasty, Medicaid patients also had worse follow-up^25^; this may make it more difficult for surgeons to identify obstacles in the recovery process that can prevent optimal outcomes. In addition, Medicaid patients have worse access to postoperative rehabilitation following rotator cuff repair.^9^ If the same is true for rehabilitation following shoulder arthroplasty, this may account for worse postoperative PROMs seen in Medicaid patients.^5^^,^^25^ Multiple studies in this review identified an association between Medicaid and increased postoperative complications,^13^^,^^43^^,^^46^ which likely accounts for the increased costs and length of stays seen in Medicaid shoulder arthroplasty patients.^13^ Sequeira et al similarly identified increased complications, reoperations, health-care utilization, and 1-year cost of care in Medicaid patients undergoing rotator cuff repair.^40^ Medicaid patients likely see increased rates of postoperative complications and health-care utilization because they exhibit a higher prevalence of comorbidities.^1^ While this association is important to explore, it is necessary to note that Medicaid patients are more likely to be disabled or low-income, meaning that this variable carries important confounding factors.^8^ Access to care and comorbidities are probably key reasons behind the association between insurance status and shoulder arthroplasty outcomes.

The high prevalence of associations between socioeconomic status and shoulder arthroplasty outcomes is likely tied to insurance status, comorbidities, and cost of care. Patients with lower socioeconomic status are more likely to rely on public insurance like Medicaid. In addition, lower-income patients are disproportionately impacted by the costs of routine postoperative visits.^39^ This may discourage these patients from engaging in their postoperative course, which limits the ability of surgeons to identify and mitigate complications. In addition, orthopedic patients with greater socioeconomic deprivation have significantly more comorbidities.^32^ This creates a prime situation for the development of postoperative complications, supported by the findings of multiple studies analyzed here.^11^^,^^26^^,^^35^^,^^38^ Other situational factors may contribute as well. For example, patients with lower income may feel increased pressure to return to work more quickly. If they return too quickly, especially to jobs that are labor intensive, this could negatively affect their recovery and increase their pain. Castle et al found that lower-income patients demonstrated worse pain and function PROMs postoperatively.^5^ Like patients with Medicaid, lower-income patients are susceptible to worse outcomes following shoulder arthroplasty likely due to the burden of routine postoperative care and higher comorbidities.

Like insurance and socioeconomic status, race was significantly associated with shoulder arthroplasty outcomes in most of the relevant studies. The results for race were slightly more varied, though. For example, Garcia et al found that Hispanic patients had a significantly lower risk of revision than White patients, and both Asian and Black patients demonstrated similar risks of revision to White patients.^14^ Garcia et al also found that Black patients had a significantly higher risk of emergency department (ED) visits postoperatively.^14^ Like Garcia, Cutler et al found that race was not predictive of revision.^10^ Multiple studies found that minority patients were more likely to have longer lengths of stay.^38^^,^^44^ Castle et al also found that Black patients had worse PROMs.^5^ The variation in results when it comes to the association between race and outcome may suggest the influence of confounding variables, such as other sociodemographic variables. The lack of consistent differences in outcomes between races could be a result of recent efforts of the medical community to minimize racial disparities for patients. Despite these efforts, some of the persisting racially based differences in outcomes may be due to unconscious biases among medical professionals. For example, unconscious racial bias in pain perception has been proven to exist despite the best intentions of the physician.^27^ If the complaints of Black patients are overlooked in routine postoperative visits and go on to become exacerbated, this could promote increased ED visits for this group of patients. Being open-minded and thoughtful about patients' concerns and complaints at postoperative visits may mitigate risks of readmission and ED visits for all patients, but especially for those prone to racial biases. Race demonstrates the most variable results in its effect on postoperative outcomes, but for those patients at risk of worse outcomes based on race, close partnership, and mutual trust between patients and physicians may promote better outcomes.

Education is an understudied variable in relation to total shoulder arthroplasty despite its probable influence over postoperative outcomes. Research has demonstrated that education level is associated with outcomes following total knee arthroplasty and total hip arthroplasty in multiple studies.^16^^,^^17^^,^^50^ Education was explicitly investigated by only two studies that found educational disparity to be associated with complications, ED visits, and instability clinically and with worse PROMs.^19^^,^^35^ One reason education may be understudied is because it is a variable that may be grouped with socioeconomic status and not investigated on its own. Among poor communities, though, those without a college education scored significantly worse on PROMs than those with a college education.^16^ Thus, it deserves independent investigation separate from socioeconomic status, given its independent role. A lower level of education may contribute to ED visits due to a lack of understanding of what warrants emergency care. In patients undergoing elective spine surgery, a college education is associated with reduced ED visits postoperatively.^31^ Orthopedic patients with less education are also more likely to have lower health literacy.^20^ This may reduce their ability to properly care for themselves postoperatively, follow postoperative instructions, and self-monitor for the development of complications. Physicians should consider taking more time to counsel and guide patients undergoing shoulder arthroplasty who are from a lower educational background. The extra effort to ensure a good understanding of postoperative instructions by the patient and their support system may help to mitigate some of the educational disparities in shoulder arthroplasty outcomes.

This study is not without limitations. Nearly all studies reported on both aTSA and rTSA together, making it impossible to investigate the role of sociodemographics on their outcomes individually. In addition, some of the studies included here were database studies, and multiple used the same databases, meaning there was likely overlap in the populations investigated here. Despite attempts to create a comprehensive search strategy, it is also possible that we did not capture all relevant studies.

## Conclusion

The majority of studies investigating the influence of insurance status, race, socioeconomic status, and education on shoulder arthroplasty outcomes found significant associations. Patients using public insurance, who are minorities, who are socioeconomically or educationally disadvantaged should all be identified by physicians as being at higher risk of poor outcomes. Physicians should partner with these patients to counsel them accordingly and to identify opportunities to improve their chances of success such as asking patients about obstacles in their recovery and reviewing rehabilitation exercises and follow-up appointments. With these findings physicians can also more closely monitor patients they identify to be vulnerable to worse outcomes to ideally prevent or catch complications earlier.

## Disclaimers:

Funding: No funding was disclosed by the authors.

Conflicts of interest: Joseph N Liu reports a relationship with Stryker Orthopaedics that includes speaking and lecture fees and with Innocoll Biotherapeutics NA Inc. that includes travel reimbursement. All the other authors, their immediate families, and any research foundation with which they are affiliated have not received any financial payments or other benefits from any commercial entity related to the subject of this article.
